# The Effect of Haplotypes in the *CETP* and *LIPC* Genes on the Triglycerides to HDL-C Ratio and Its Components in the Roma and Hungarian General Populations

**DOI:** 10.3390/genes11010056

**Published:** 2020-01-03

**Authors:** Peter Piko, Szilvia Fiatal, Nardos Abebe Werissa, Bayu Begashaw Bekele, Gabor Racz, Zsigmond Kosa, Janos Sandor, Roza Adany

**Affiliations:** 1 MTA-DE Public Health Research Group, Public Health Research Institute, University of Debrecen, 4028 Debrecen, Hungary; piko.peter@sph.unideb.hu (P.P.); nardos.abebe@sph.unideb.hu (N.A.W.); 2 Department of Preventive Medicine, Faculty of Public Health, University of Debrecen, 4028 Debrecen, Hungary; fiatal.szilvia@sph.unideb.hu (S.F.); racz.gabor@sph.unideb.hu (G.R.); sandor.janos@sph.unideb.hu (J.S.); 3 WHO Collaborating Centre on Vulnerability and Health, University of Debrecen, 4028 Debrecen, Hungary; 4 Doctorial School of Health Sciences, University of Debrecen, Debrecen 4028, Hungary; bayu.begashaw@sph.unideb.hu; 5 Department of Health Visitor Methodology and Public Health, Faculty of Health, University of Debrecen, 4400 Nyíregyháza, Hungary; kosa.zsigmond@foh.unideb.hu

**Keywords:** single nucleotide polymorphism, triglyceride, high-density lipoprotein cholesterol, TG/HDL-C ratio, Roma, Hungarian general, *CETP*, *LIPC*, haplotype, cardiometabolic risk

## Abstract

Background: The triglycerides (TG) to high-density lipoprotein (HDL)-cholesterol (HDL-C) ratio (TG/HDL-C) is a well-known predictor for cardiovascular diseases (CVDs) with great heritability background. The cholesteryl ester transfer protein (*CETP*) and hepatic lipase (*LIPC*) gene affect TG/HDL-C ratio. This study aims to explore the association between haplotypes (H) in *CETP* (based on 5 single nucleotide polymorphisms (SNPs)) and *LIPC* (based on 6 SNPs) genes and the TG/HDL-C ratio and its components, among Roma and Hungarian general populations. Methods: The prevalence of haplotypes and their effect on HDL-C, TG and TG/HDL-C ratio were calculated in both populations and compared. Results: Ten haplotypes in *CETP* and 6 in *LIPC* gene were identified. Three haplotypes in *CETP* and 3 in *LIPC* have significant effect on HDL-C level, whereas two in *CETP* and 3 in *LIPC* on TG level. The H6 in *CETP* (β = 0.52, *p* = 0.015; odds ratio (OR) = 1.87, *p* = 0.009) and H5 in *LIPC* (β = 0.56, *p* < 0.001; OR = 1.51, *p* = 0.002) have a significant increasing effect on TG/HDL-C ratio and have shown higher prevalence among the Roma, as compared to Hungarian general population. The H2 in the *CETP* gene has a decreasing effect on the TG/HDL-C ratio (OR = 0.58, *p* = 0.019) and is significantly less frequent among the Roma. Conclusions: Accumulation of harmful haplotypes in *CETP* and *LIPC* genes might have a role in the elevated TG/HDL-C ratio in the Roma population, which contributes to a higher risk in the development of cardiovascular diseases.

## 1. Introduction

Cardiovascular diseases (CVDs) are the leading cause of death globally. More people die from CVDs annually than from any other cause. Statistical data from 2016 show that CVDs were, by far, the leading cause of death in the 28 member states of the European Union (EU-28). The standardized death rate for circulatory diseases was 737.5 deaths per 100,000 inhabitants in Hungary (5th worst among the EU-28 countries), which was twice that of the EU-28 average (358.3 deaths per 100,000) [1].

Roma are the largest ethnic minority population of Europe and one of the main subjects of ethnicity-based studies. Approximately 10–12 million Roma live in the continent, mainly (aggregated) in Central Eastern and Southern European countries (in Bulgaria, Hungary, Slovakia and Romania) [2]. As reported by the latest census in 2011, the representation of the Roma in Hungary was an estimated 3.2%, however, their real representation is much higher, approximately 8–10% of the total population [3,4]. They are mostly concentrated in the Northeast region of the country, where they frequently live in segregated colonies with severe environmental problems, such as the lack of sewage and gas mains, garbage deposits, waterlogged soil and lack of water mains [5]. They suffer from poor health and limited access to healthcare [6]. Studies on the Roma population furthermore face many challenges in data collection and methodology [7,8].

A number of studies have investigated the prevalence of risk factors of CVDs (e.g., obesity, hypertension, smoking, lipids and lipoprotein profile, etc.) among Roma and found it to be higher than in the general population [9,10,11,12]. Comparative studies on the risk profile for Roma adults indicate that the Roma population has significantly lower high-density lipoprotein cholesterol (HDL-C) levels, as well as no or slightly elevated TG levels in comparison to the majority population, irrespective of the country where they live [13,14,15].

The atherogenic lipoprotein profile of the plasma level is one of the most important risk factors/predictors for the development of cardiovascular diseases. It is characterized by a low level of high-density lipoprotein cholesterol (HDL-C) and increased level of triglyceride (TG) [16]. The high-density lipoprotein is the key component involved in reverse cholesterol transport and the transfer of cholesteryl esters between lipoproteins [17]. The level of HDL-C is inversely associated with the risk of coronary heart diseases and is interpreted as a key indicator of predicting CVD risk [18]. The elevated level of TG is also considered to be an independent risk marker for the development of CVDs [19].

The TG and HDL-C levels are interrelated; the ‘triglycerides to HDL-C ratio’ (TG/HDL-C) summarizes the combined atherogenic effect of these levels and can be used as a better predictor for the development of CVDs. The predictive value for CVDs of the TG/HDL-C ratio was significantly higher compared to TG and HDL-C individually [20]. The TG/HDL-C ratio of 1.0 was concluded as an optimal cut-off point and its elevated value was suggested as a predictor and an early marker for increased cardiometabolic risk (CMR) by Qurat et al. [21]. In addition, da Luz et al. [20] demonstrated that a TG/HDL-C ratio higher than 4.0 might predict extensive risk for development of coronary diseases (CDs).

Our previous study showed that genetic factors contribute to the higher prevalence of reduced HDL-C levels among Roma [22,23]. However, the genetic background concerning the TG level or TG/HDL-C ratio has never been investigated on the Roma population.

The genetic determination of the HDL-C and TG levels is high (46% and 36%, respectively) [24]. Frequent polymorphisms in the genes of cholesteryl ester transfer protein (*CETP*) [25,26,27,28,29] and hepatic lipase (*LIPC*) [25,30,31,32] play an important role in lipid metabolism by catalyzing the exchange of cholesterol and other lipids between circulating lipoprotein classes [33], which have been reported to be significantly associated with HDL-C and TG levels in multiple populations.

The major goal of our study is to identify haplotype blocks in the *CETP* and *LIPC* genes and estimate the effect of them on the TG/HDL-C ratio, as well as HDL-C and TG levels separately. Our other aim is to compare the frequencies of these haplotypes between the Roma and Hungarian general populations to provide additional information on the presence of genetic factors behind the cardiovascular risk associated with the lipid profile in both populations.

## 2. Materials and Methods

### 2.1. Study Design

Our study involved subjects of representative samples investigated during/over the course of recent cross-sectional surveys [15,34]. The subjects included 757 Hungarian Roma individuals living in segregated colonies in the Northeast of Hungary, where the Roma are concentrated, and 1783 individuals from the Hungarian general population.

All procedures performed in studies involving human participants were in accordance with the ethical standards of the institutional and/or national research committee and with the 1964 Helsinki declaration and its later amendments or comparable ethical standards. This study was approved by the Ethical Committee of the University of Debrecen, Medical Health Sciences Centre (reference No. 2462-2006) and by the Ethical Committee of the Hungarian Scientific Council on Health (reference Nos. NKFP/1/0003/2005 and 8907-O/2011-EKU). This article does not contain any studies with animals performed by any of the authors.

### 2.2. Sample Populations

#### 2.2.1. Roma Living in Segregated Colonies

Participants were gathered from the Northeast region of Hungary (Hajdú-Bihar and Szabolcs-Szatmár-Bereg counties), where the majority of Hungarian Roma colonies can be found, using a stratified multistage sampling method. The details of sampling methodology and the data collected are described in our previous paper [15]. As part of this health examination survey, each participant’s medical history and socio-demographic characteristics were recorded, and the participants also underwent physical examinations. Blood samples were taken for laboratory and genotype investigations. The present study used 757 samples, as well as complete clinical records of 20–64-year-old Roma adults, where available.

#### 2.2.2. Hungarian General Population

A population-based disease monitoring system, the General Practitioners’ Morbidity Sentinel Stations Programme (GPMSSP), provided the Hungarian reference sample [34,35]. Samples were drawn from the population of counties participating in the GPMSSP. The methods of sampling applied and survey data collected are described in the Hungarian Metabolic Syndrome Survey (HMSS) [35]. As part of a health examination survey, medical histories and socio-demographic characteristics were recorded and physical examinations were carried out for each participant. Blood samples were taken for laboratory tests and for DNA isolation. The present study used DNA samples from 1783 adults aged 20–60 with complete records to create the reference dataset. The sample is representative of the Hungarian adult population in terms of geographic, age and gender distributions.

### 2.3. DNA Isolation, Selection of SNPs and Genotyping

DNA was isolated using a MagNA Pure LC system (Roche Diagnostics, Basel, Switzerland) with a MagNA Pure LC DNA Isolation Kit—Large Volume according to the manufacturer’s instructions. Extracted DNA was eluted in 200 µL MagNA Pure LC DNA Isolation Kit—Large Volume elution buffer.

A systematic literature review on the PubMed, HuGE Navigator and Ensembl databases was conducted to identify single nucleotide polymorphisms (SNPs) in *CETP* and *LIPC* genes, which are most strongly associated with cholesterol metabolism. The literature search resulted in the selection of 5 SNPs in *CETP* and 6 SNPs in the *LIPC* gene. The genotyping was conducted by the Mutation Analysis Core Facility at the Karolinska University Hospital, Sweden.

Genotyping was performed on a MassARRAY platform (Sequenom Inc., San Diego, CA, USA) with iPLEX Gold chemistry. Validation, concordance analysis and quality control were conducted by the facility according to their protocols. Successful genotyping was obtained in 2518 DNA samples (746 Roma and 1772 Hungarian general samples).

More details on the study design, sample populations, selection process of SNPs and genotyping are described in our previous research paper [22].

### 2.4. Statistical Analyses

Statistical tests were conducted with the SNPStats online tool (http://bioinfo.iconcologia.net/SNPstats), IBM SPSS (version 22, IBM Company, Armonk, NY, USA) and Haploview (version 4.2, Broad Institute, Cambridge, MA, USA). Linkage disequilibrium structure for both genes was created by Haploview software. Mann–Whitney U tests were used to compare the age, body mass index (BMI), systolic and diastolic blood pressure, fasting glucose, TG and HDL-C level and TG/HDL-C ratio of the populations. Sex distribution, lipid-lowering, antihypertensive and antidiabetic treatment, HDL-C (<1.03 mmol/L in male and <1.29 mmol/L in female), TG (≥1.7 mmol/L) and TG/HDL status (TG/HDL ratio is ≥1 [21] for elevated and >4 [20] for highly elevated) were compared by χ^2^ tests. The existence of the Hardy–Weinberg equilibrium (HWE) and the differences of allele frequencies for all SNP variants between the two populations were evaluated by χ^2^ tests. The SNPs haplotype block analyses were estimated via the expectation maximization algorithm carried out by the SNPStats online tool [36].

To avoid effects that are due to ethnicity related factors (e.g., environment and culture), the two populations were studied together (Roma and Hungarian general) in a combined population, and then ethnicity was used as covariate in the models. All models were adjusted by relevant covariates (e.g., ethnicity, sex, age, BMI, systolic and diastolic blood pressure, fasting glucose level, antihypertensive, antidiabetic and lipid-lowering treatment) to avoid errors due to multicollinearity.

Generally, the conventional *p* threshold of 0.05 was used, and we also applied the Bonferroni correction to generate *p*-values for multiple modeling calculations in which the number of independent SNPs were defined by using the SNPs nap web-based tool [37] (considered in the case of both genes; i.e., 4 in the *CETP* and 2 in the *LIPC*). After adjustment the *p*-values that were <0.0125 were considered to be significant for the analyses of the effect of *CETP* haplotypes and <0.025 for the analyses of the effect of *LIPC* haplotypes.

## 3. Results

### 3.1. Characteristics of the Study Populations

Samples without full geno- and phenotype data were excluded from the analyses. In total, 613 Hungarian Roma participants (HR) and 1494 individuals from the general population (HG) were included in the analyses. Details on population characteristics are listed in Table 1.

### 3.2. Haplotypes in the CETP and LIPC Genes and Their Linkage Disequilibrium Structure and Frequencies in the Roma and Hungarian General Populations

**Figure 1:**
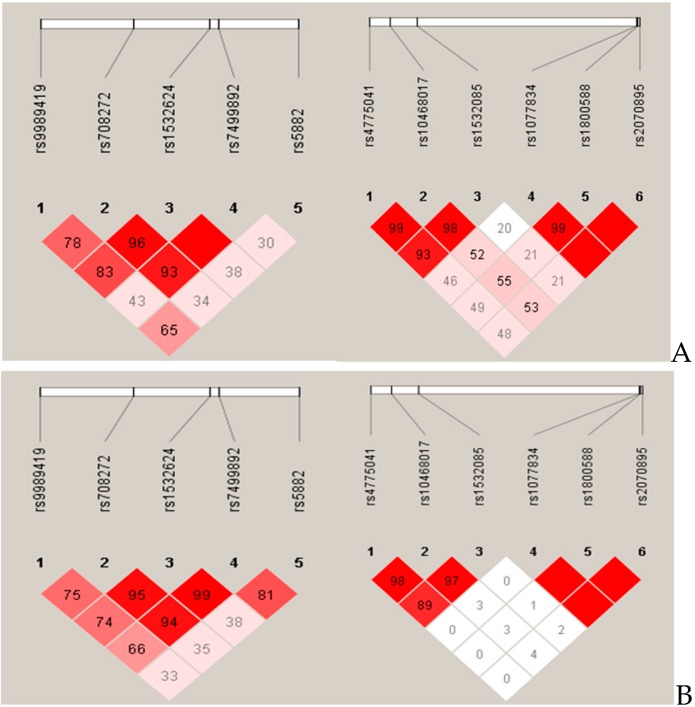
Linkage disequilibrium structure of the 5 studied single nucleotide polymorphisms (SNPs) in *CETP* and 6 SNPs in *LIPC* genes in Hungarian Roma (**A**) and general populations (**B**).

Haplotype analysis involved different combinations of the 5 SNPs in *CETP* (rs1532624, rs5882, rs708272, rs7499892 and rs9989419) and 6 SNPs in *LIPC* genes (rs10468017, rs1077834, rs1532085, rs1800588, rs2070895, rs4775041). For the results of the linkage disequilibrium structure analysis, see Figure 1.

The blocks were formed by the SNPs of the *CETP* and *LIPC* genes. The numbers above the map show the rs numbers of SNPs. The color scheme is a standard Haploview color scheme (white D′ < 1 and LOD < 2, shades of pink/red: D′ < 1 and LOD ≥ 2, and bright red D′ = 1 and LOD ≥ 2). Numbers in the squares are D′ values.

We identified 10 haplotype blocks in the *CETP* and six in *LIPC* genes, the prevalence of which had been higher than 1% in the combined population (R and HG together). A total of 8 out of 10 in the haplotypes blocks in the case of *CETP* (H1–H5 and H8–H10) and 4 out of 6 in *LIPC* (H1 and H4–H6) showed significant difference in prevalence between the study populations (see Table 2 and Table 3). The H8*_CETP_* occurs almost exclusively in the Roma population (HR: 7.28% vs. HG: 0.14%; *p* < 0.001).

### 3.3. Association of Haplotypes in CETP and LIPC Genes with HDL-C and TG Levels in Combined Population

The frequency of the most prevalent haplotypes of the genes investigated in the combined population (H1*_CETP_*: AGACG and H1*_LIPC_*: CTGCGG) were used as references for comparative analysis of their relationship with HDL-C and TG, both as continuous and as binary outcomes.

H3*_CETP_* (β_HDL-C_ = −0.05, *p* = 0.016; OR_HDL-C_ = 1.34, *p* = 0.040 and β_TG_ = −0.16, *p* = 0.033) and H8*_CETP_* (_βHDL-C_ = −0.14 *p* = 0.001; OR_HDL-C_ = 2.60, *p* = 0.002 and β_TG_ = −0.33, *p* = 0.032; OR_TG_ = 0.50, *p* = 0.026) have at least a nominally significant lipid-lowering effect on both outcomes. The prevalence of H3*_CETP_* in the Hungarian general population (HG: 15.05% vs. HR: 11.45%, *p* = 0.015), and the prevalence of H8*_CETP_* in the Roma population (HG: 0.14% vs. HR: 7.28%, *p* < 0.001) were found to be significantly higher in comparison with the other.

The H2*_LIPC_* (β_HDL-C_ = 0.05, *p* = 0.003; OR_HDL-C_ = 0.74, *p* = 0.006 and β_TG_ = 0.16, *p* = 0.005, OR_TG_ = 1.30, *p* = 0.025) and H3*_LIPC_* (β_HDL-C_ = 0.07, *p* = 0.001 and β_TG_ = 0.21, *p* = 0.004; OR_TG_ = 1.57, *p* = 0.002) have a significant effect on both lipid parameters, and their prevalence did not differ significantly between the study groups. H5*_LIPC_* (β_HDL-C_ = 0.09, *p* = 0.004; OR_HDL-C_ = 0.50, *p* = 0.005) was significantly associated only with HDL-C, and its prevalence was significantly higher in the HG population (HG: 5.48% vs. HR: 2.81%, *p* = 0.006). H6*_LIPC_* (β_TG_ = 0.45, *p* < 0.001; OR_TG_ = 2.39, *p* = 0.001) showed association only with TG and was significantly more frequent among Roma (HG: 2.08% vs. HR: 6.63%, *p* < 0.001). More details on the effect of haplotypes on HDL-C and TG levels are shown in Table 4 (for the *CETP* gene) and Table 5 (for the *LIPC* gene).

### 3.4. Association of Haplotypes in CETP and LIPC Genes with TG/HDL-C Ratio

H5*_CETP_* (β = 0.56, *p* < 0.001; ORCMR = 1.51, *p* = 0.002 and ORCD = 1.59, *p* = 0.017) and H6*_LIPC_* (β = 0.52, *p* = 0.015; ORCMR = 1.87, *p* = 0.009) showed significant association with elevated TG/HDL-C ratio and their prevalence was significantly higher among the Hungarian Roma, as compared with the Hungarian general population (H5*_CETP_*: HG: 11.60% vs. HR: 15.07%, *p* = 0.019 and H6*_LIPC_*: 6.63% vs. 2.08%, *p* < 0.001) as showed in Table 6. The H3*_LIPC_* (OR_CD_ = 1.57, *p* = 0.024) had an increasing effect on highly elevated TG/HDL-C ratio but its prevalence did not differ significantly between the study groups (HR: 14.25% vs. HG: 13.63%, *p* = 0.470).

It was found that H2*_CETP_* reduces the possibility of having a highly elevated TG/HDL-C ratio (OR_CD_ = 0.58, *p* = 0.019) and it was less frequent among the Hungarian Roma population (14.24% vs. 21.78%, *p* < 0.001). More details on the effect of haplotypes on the TG/HDL-C ratio are found in Table 6 (for *CETP* gene) and Table 7 (for *LIPC* gene).

## 4. Discussion

Various studies were conducted to define the prevalence of cardiovascular risk factors among Roma and it was found to be highly increased among them [9,10,11,12]. The elevated TG/HDL-C ratio is one of the most important risk predictors with a high heritability background [24], and it combines the risk represented by reduced HDL-C and elevated TG levels, which are predictors of the early onset of cardiovascular diseases [20,21]. Our study was designed to identify haplotypes in *CETP* and *LIPC* genes (based on 5 SNPs in the *CETP* and 6 SNPs in the *LIPC* gene) and compare their prevalence between the Hungarian general and Roma populations, as well to analyze the association between haplotypes and the TG/HDL-C ratio and its components (HDL-C and TG).

Ten haplotypes in the *CETP* and 6 in the *LIPC* gene were identified. Eight in the *CETP* (H1–H5 and H8–H10) and 4 in the *LIPC* (H1 and H4–H6) showed a frequency significantly different between the two study populations. Out of the 16 studied haplotypes, the H8*_CETP_* was almost exclusive to the Roma population (its frequency was 7.28% in the Roma vs. 0.14% in the general population).

The most frequent haplotypes in the combined population (H1*_CETP_*: AGACG; H1*_LIPC_*: CTGCGG) were used as references during the analyses. Three haplotypes in *CETP* (H3, H5 and H8) and 3 in *LIPC* (H2, H3 and H5) were shown to have a significant effect on HDL-C, and 2 in *CETP* (H3 and H8) and 3 in *LIPC* (H2, H3 and H6) on TG level.

Both H5*_CETP_* and H6*_LIPC_* turned out to have a significant effect on elevating TG/HDL-C ratio and their frequency was significantly higher among the Roma population. H3*_LIPC_* also had an effect on highly elevated TG/HDL-C ratio, but its prevalence did not differ significantly between the study groups. H2*_CETP_* showed a reducing effect on the risk of having a highly elevated TG/HDL-C ratio and it was significantly less frequent among the Roma. In our previous study we confirmed that the effect size on HDL-C level of the 5 SNPs in the *CETP* and 6 in the *LIPC* genes we investigated in our present study did not differ significantly between the Hungarian general and Roma population [21], so we can conclude that the combination of these haplotypes may contribute to the higher prevalence of elevated TG/HDL-C ratio among Roma.

The elevated TG/HDL-C ratio has been shown to be closely associated with the onset of cardiovascular disease, which leads to shorter life expectancy and premature death [38]. The significantly higher prevalence of haplotypes with TG/HDL-C ratio raising effect (H5*_CETP_* and H6*_LIPC_*) and significantly less frequent prevalence of the haplotype with TG/HDL-C ratio lowering effect (H3*_LIPC_*) may contribute to an elevated risk for the development of cardiovascular diseases among Roma.

Understanding the genetic background of the elevated TG/HDL-C ratio can help to identify those molecules involved in lipid metabolism, especially in cholesterol transport that can later be used to develop targeted gene therapies. There is currently a limited number of studies on the effect of haplotypes in *CETP* [39,40] and *LIPC* [41] genes on the HDL-C level, but no study has investigated their effect on TG level and/or TG/HDL ratio. The *LIPC* gene codes the synthesis of hepatic lipase enzyme, which catalyzes the conversion of intermediate-density lipoprotein to low-density lipoprotein, and assists in the transport of HDL-C carrying the triglycerides and cholesterols from the blood to the liver, and also play a role on hydrolysis of TGs [42]. The rs1800588 (in LIPC gene), which we have analyzed in our study, has been previously described as one of the modulators of the HDL-cholesterol response to statin treatment [43]. Currently, lipid treatment targeting a LIPC gene product is unknown, so our finding may open new opportunities in preventive medication.

The *CETP* transfers cholesteryl esters from HDL-C to apolipoprotein B-containing particles in exchange for triglyceride, thereby reducing the concentration of HDL-C. *CETP* inhibitors have proven to be effective in achieving both a reduction in low density lipoprotein cholesterol and an increase in HDL-C [44]. The cholesteryl ester transfer protein is suggested as a possible novel target for raising HDL-C and inhibiting atherosclerosis [29], although on the basis of the results obtained in large scale cardiovascular clinical outcome trials, the future of *CETP* inhibition as a potential therapeutic option for reducing major cardiovascular events is currently uncertain [45].

Several haplotypes in the *CETP* gene have been described in the literature, which have an effect on the lipid-modifying response to various statin therapies [46,47]. With the recognition of these gene alterations, personalized gene therapies will be available to the carriers. Knowledge of these therapeutically relevant haplotypes would have many individual and societal benefits by providing targets for personalized medication, as well as precision prevention medication [48]. Furthermore, it is a well-known fact that lipid and carbohydrate metabolism are strongly related to each other. Numerous studies have described that TG/HDL-C ratio has an impact on insulin resistance (HOMA-IR) [49,50,51,52] in addition to its cardiovascular risk effect. The prevalence of raised fasting glucose levels and T2DM is higher among the Roma in comparison to the majority population [14,53]. This phenotypic characteristic might be explained by the connection between TG/HDL-C ratio and insulin resistance, but further research would be required to prove it.

Our current study obviously had limitations. Accurate ethnic identification is a common challenge of studies such as ours. Roma ethnicity was self-reported and Roma samples were collected from Northeast Hungary, where these individuals are accumulated in segregated colonies. Therefore, this sample cannot be interpreted as a representative sample for the whole Hungarian Roma population. Moreover, the presence of Roma ethnicity is estimated to be more than 8% in the general Hungarian population, so it is possible that their inclusion resulted in a slight underestimation of the differences between the two populations. Some factors that were not taken into consideration in the present study (epigenetic factors, rare or structural variants, gene–environmental and gene–gene interactions) also have an effect on the outcomes we investigated, and they could modify the results. The present analyses were adjusted for relevant covariates; however, several environmental and lifestyle factors (such as physical inactivity and poor diet) can modify susceptibility to the trait. In addition, further genetic replication study in an independent Roma population sample is needed to validate our results.

## 5. Conclusions

In conclusion, we found that haplotypes formed from 5 SNPs in *CETP* and 6 SNPs in *LIPC* genes were at least nominal significantly associated with TG/HDL-C ratio and its components. Two of the haplotypes (H5*_CETP_* and H6*_LIPC_*) indicating higher risk for the development of CVDs through elevated TG/HDL-C ratio were significantly more frequent in the Roma population, whereas the H2*_CETP_* with a cardiovascular protective effect, was significantly less frequent among Roma people in comparison to the general Hungarian population.

These findings confirm that genetic factors are the underlying cause of an elevated TG/HDL-C ratio and they may contribute to the increased risk for the development of cardiovascular disease in the Roma population.

We believe that our findings will contribute to the identification of people with an elevated cardiometabolic risk (through the accumulation of harmful haplotypes in *CETP* and *LIPC* genes) and can be used even for the development of a screening tool aimed at elevated cardiometabolic risk.

## Figures and Tables

**Table 1:** Demographic characteristics of the study populations.

	Hungarian General (*n* = 1494)	Roma (*n* = 613)	
	Mean (95% CI)		*p*-Value
Age (year)	44.16 (43.53–44.78)	40.3 (39.39–41.21)	<0.001
Body mass index (kg/m^2^)	27.43 (27.15–27.70)	27.47 (26.64–28.30)	0.898
Systolic blood pressure (mmHg)	126.81 (125.95–127.66)	125.21 (123.67–126.76)	0.059
Diastolic blood pressure (mmHg)	80.26 (79.79–80.72)	78.43 (77.63–79.23)	<0.001
Fasting glucose level (mmol/L)	4.83 (4.74–4.92)	5.44 (5.29–5.59)	<0.001
High-density lipoprotein-cholesterol (HDL-C) level (mmol/L)	1.43 (1.40–1.45)	1.21 (1.18–1.24)	<0.001
Triglyceride (TG) level (mmol/L)	1.63 (1.55–1.72)	1.61 (1.50–1.71)	0.760
Triglycerides to HDL-C ratio (TG/HDL-C) ratio	1.48 (1.35–1.61)	1.67 (1.47–1.86)	0.115
	Prevalence (%)		*p*-Value
Sex (female/male)	52.7/47.3	61.7/38.3	<0.001
Antihypertensive treatment	29.90	25.40	0.042
Lipid-lowering treatment	13.90	10.80	0.050
Antidiabetic treatment	5.40	5.20	0.901
Reduced HDL-C level ^1^	28.20	53.00	<0.001
Elevated TG level ^2^	30.32	28.71	0.463
Elevated TG/HDL-C ratio (≥1) ^3^	42.44	53.83	<0.001
Highly elevated TG/HDL-C ratio (>4) ^4^	5.09	5.87	0.465

^1^ Reduced HDL-C level: <1.03 mmol/L in male and <1.29 mmol/L in female. ^2^ Elevated TG level: ≥1.7 mmol/L in both sexes. ^3^ TG/HDL-C ratio cut-off for increased cardiometabolic risk (CMR) by Qurat et al. [21]. ^4^ TG/HDL-C ratio cut-off for extensive coronary disease (CD) risk by da Luz et al. [20].

**Table 2:** The frequency of haplotypes in *CETP* gene in the combined sample, as well as in Roma and Hungarian general populations.

Haplotypes	Rs1532624	Rs5882	Rs708272	Rs7499892	Rs9989419	Frequency in Combined Population (*n* = 2107)	Frequency in Hungarian General Population (*n* = 1494)	Frequency in Roma Population (*n* = 613)	*p*-Value
**H1**	**A**	**G**	**A**	**C**	**G**	20.57%	17.32%	**28.29%**	**<0.001**
**H2**	**A**	**A**	**A**	**C**	**G**	19.55%	**21.78%**	14.24%	**<0.001**
**H3**	**C**	**A**	**G**	**C**	**A**	13.98%	**15.05%**	11.45%	**0.015**
**H4**	**C**	**A**	**G**	**C**	**G**	13.69%	**14.79%**	11.09%	**0.015**
**H5**	**C**	**A**	**G**	**T**	**A**	12.63%	11.60%	**15.07%**	**0.019**
H6	C	G	G	C	A	5.55%	5.95%	4.60%	0.170
H7	A	A	A	C	A	2.77%	2.92%	2.43%	0.467
**H8**	**C**	**G**	**G**	**T**	**G**	2.26%	0.14%	**7.28%**	**<0.001**
**H9**	**C**	**G**	**G**	**C**	**G**	2.60%	**3.04%**	1.56%	**0.039**
**H10**	**C**	**A**	**G**	**T**	**G**	2.43%	**2.86%**	1.41%	**0.038**

Haplotypes with significantly different frequency in the two populations (*p* < 0.05) and their frequency with the higher value are highlighted in bold.

**Table 3:** The frequency of haplotypes in *LIPC* gene in the combined sample, as well as in Roma and Hungarian general populations.

Haplotypes	Rs10468017	Rs1077834	Rs1532085	Rs1800588	Rs2070895	Rs4775041	Frequency in Combined Population (*n* = 2107)	Frequency in Hungaria*n* General Populatio*n* (*n* = 1494)	Frequency in Roma Population (*n* = 613)	*p*-Value
**H1**	**C**	**T**	**G**	**C**	**G**	**G**	44.41%	**48.38%**	34.97%	**<0.001**
H2	T	T	A	C	G	C	21.08%	20.40%	22.70%	0.073
H3	C	C	G	T	A	G	13.81%	13.63%	14.25%	0.470
**H4**	**C**	**T**	**A**	**C**	**G**	**G**	10.20%	7.23%	**17.24%**	**<0.001**
**H5**	**T**	**C**	**A**	**C**	**G**	**G**	4.69%	**5.48%**	2.81%	**0.006**
**H6**	**C**	**C**	**A**	**T**	**A**	**G**	3.43%	2.08%	**6.63%**	**<0.001**

Haplotypes with significantly different frequency in the two populations (*p* < 0.05) and their frequency with the higher value are highlighted in bold.

**Table 4:** The effect of haplotypes in *CETP* gene on high-density lipoprotein cholesterol (HDL-C) and triglyceride (TG) levels in the combined study population (Hungarian Roma and Hungarian general together). The association was evaluated under adjusted models (ethnicity, sex, age, body mass index, systolic and diastolic blood pressure, fasting glucose level, antihypertensive, antidiabetic and lipid-lowering treatment).

Haplotypes	HDL-C				TG			
	β (95% CI)	*p*-Value	OR (95% CI)	*p*-Value	β (95% CI)	*p*-Value	OR (95% CI)	*p*-Value
H1	reference	---	reference	---	reference	---	reference	---
H2	0.02 (−0.01–0.06)	0.220	0.88 (0.67–1.16)	0.370	−0.01 (−0.15–0.13)	0.890	0.94 (0.71–1.24)	0.650
**H3**	**−0.05** **(−0.09)–(−0.01)**	0.016 *	**1.34** **(1.01–1.76)**	0.040 *	**−0.16** **(−0.30)–(−0.01)**	0.033 *	0.85 (0.63–1.14)	0.270
H4	−0.01 (−0.06–0.03)	0.470	0.91 (0.68–1.20)	0.490	−0.03 (−0.18–0.11)	0.650	1.02 (0.76–1.36)	0.900
**H5**	**−0.11** **(−0.16)–(−0.07)**	<0.001 **	**1.74** **(1.32–2.30)**	<0.001 **	−0.01 (−0.16–0.13)	0.840	0.92 (0.68–1.23)	0.560
H6	0.01 (−0.06–0.07)	0.880	0.86 (0.55–1.34)	0.500	0.16 (−0.08–0.39)	0.180	0.99 (0.63–1.57)	0.980
H7	0.04 (−0.05–0.13)	0.370	0.93 (0.50–1.72)	0.810	−0.03 (−0.36–0.29)	0.850	0.75 (0.37–1.53)	0.430
**H8**	**−0.14** **(−0.22)–(−0.06)**	0.001 **	**2.60** **(1.43–4.72)**	0.002 **	**−0.33** **(−0.62)–(−0.03)**	0.032 *	**0.50** **(0.27–0.92)**	0.026 *
H9	−0.07 (−0.17–0.03)	0.140	1.04 (0.50–2.13)	0.920	−0.31 (−0.68–0.05)	0.095	0.47 (0.19–1.12)	0.087
H10	−0.07 (−0.16–0.02)	0.130	1.80 (0.98–3.30)	0.058	−0.09 (−0.41–0.23)	0.570	1.11 (0.60–2.04)	0.740

At least nominally significant associations between haplotypes and lipid levels (cut-off for HDL-C: <1.03 mmol/L in male and <1.29 mmol/L in female; TG: <1.7 mmol/L in both sexes) are highlighted in bold. * Significant *p*-values without Bonferroni correction. ** Significant *p*-values with Bonferroni correction.

**Table 5:** The effect of haplotypes in *LIPC* gene on high-density lipoprotein cholesterol (HDL-C) and triglyceride (TG) in combined study population (Hungarian Roma and Hungarian general together). The association was evaluated under adjusted models (ethnicity, sex, age, body mass index, systolic and diastolic blood pressure, fasting glucose level, antihypertensive, antidiabetic and lipid-lowering treatment).

Haplotypes	HDL-C				TG			
	β (95% CI)	*p*-Value	OR (95% CI)	*p*-Value	β (95% CI)	*p*-Value	OR (95% CI)	*p*-Value
H1	reference	---	reference	---	reference	---	reference	---
**H2**	**0.05** **(0.01–0.08)**	0.004 **	**0.75** **(0.60–0.92)**	0.007 **	**0.16** **(0.05–0.27)**	0.005 **	**1.30** **(1.03–1.63)**	0.025 *
**H3**	**0.07** **(0.03–0.11)**	0.001 **	0.78 (0.59–1.03)	0.079	**0.21** **(0.07–0.35)**	0.004 **	**1.57** **(1.18–2.10)**	0.002 **
H4	0.03 (−0.01–0.07)	0.160	0.97 (0.73–1.29)	0.840	0.04 (−0.11–0.19)	0.590	0.98 (0.71–1.35)	0.890
**H5**	**0.09** **(0.03–0.15)**	0.005 **	**0.50** **(0.31–0.81)**	0.005 **	0.16 (−0.06–0.39)	0.150	1.23 (0.78–1.95)	0.380
**H6**	0.05 (−0.02–0.13)	0.170	0.91 (0.56–1.46)	0.680	**0.45** **(0.20–0.69)**	<0.001 **	**2.39** **(1.46–3.92)**	0.001 **

At least nominally significant associations between haplotypes and lipid levels (cut-off for HDL-C: <1.03 mmol/L in male and <1.29 mmol/L in female; TG: <1.7 mmol/L in both sexes) are highlighted in bold. * Significant *p*-value without Bonferroni correction. ** Significant *p*-values with Bonferroni correction.

**Table 6:** The effect of haplotypes in *CETP* gene on triglycerides to HDL-C ratio (TG/HDL-C ratio) in combined population (Hungarian Roma and Hungarian general together). The association was evaluated under adjusted models (ethnicity, sex, age, body mass index, systolic and diastolic blood pressure, fasting glucose level, antihypertensive, antidiabetic and lipid-lowering treatment).

Haplotypes	β (95% CI)	*p*-Value	OR_CMR_^a^ (95% CI)	*p*-Value	OR_CD_^b^ (95% CI)	*p*-Value
H1	reference	---	reference	---	reference	---
**H2**	−0.12 (−0.36–0.13)	0.350	0.82 (0.64–1.06)	0.130	**0.58 (0.37–0.91)**	0.019 *
H3	−0.02 (−0.27–0.22)	0.870	0.92 (0.71–1.18)	0.510	0.96 (0.62–1.49)	0.860
H4	0.01 (−0.24–0.25)	0.970	0.98 (0.76–1.26)	0.850	1.05 (0.69–1.58)	0.820
**H5**	**0.56 (0.31–0.82)**	<0.001 **	**1.51 (1.16–1.95)**	0.002 **	**1.59 (1.09–2.33)**	0.017 *
H6	0.24 (−0.17–0.65)	0.250	1.06 (0.70–1.59)	0.790	1.25 (0.66–2.34)	0.500
H7	−0.24 (−0.79–0.31)	0.400	0.80 (0.45–1.40)	0.430	0.98 (0.38–2.51)	0.960
H8	0.14 (−0.36–0.64)	0.580	1.27 (0.74–2.17)	0.390	0.88 (0.38–2.02)	0.760
H9	−0.05 (−0.67–0.56)	0.870	0.69 (0.36–1.32)	0.260	0.61 (0.15–2.54)	0.500
H10	0.14 (−0.40–0.68)	0.610	1.00 (0.58–1.75)	0.990	1.37 (0.60–3.10)	0.450

At least nominally significant association between haplotypes and TG/HDL-C ratio is highlighted in bold. ^a^ TG/HDL-C ratio cut-off for increase cardiometabolic risk (CMR) by Qurat et al. [21]. ^b^ TG/HDL-C ratio cut-off for extensive coronary disease (CD) risk by da Luz et al. [20]. * Significant *p*-values without Bonferroni correction. ** Significant *p*-values with Bonferroni correction.

**Table 7:** The effect of haplotypes in *LIPC* gene on triglycerides to HDL-C ratio (TG/HDL-C ratio) in combined population (Hungarian Roma and Hungarian general together). The association was evaluated under adjusted models (ethnicity, sex, age, body mass index, systolic and diastolic blood pressure, fasting glucose level, antihypertensive, antidiabetic and lipid-lowering treatment).

Haplotypes	β (95% CI)	*p*-Value	OR_CMR_^a^ (95% CI) (Cut-Off: 1)	*p*-Value	OR_CD_^b^ (95% CI) (Cut-Off: 4)	*p*-Value
H1	reference	---	reference	---	reference	---
H2	0.07 (−0.12–0.26)	0.480	1.00 (0.82–1.21)	0.970	1.23 (0.89–1.70)	0.220
**H3**	0.05 (−0.19–0.29)	0.690	0.99 (0.77–1.27)	0.940	**1.57 (1.06–2.32)**	0.024 **
H4	−0.07 (−0.33–0.18)	0.570	0.81 (0.62–1.06)	0.130	1.15 (0.72–1.83)	0.560
H5	−0.15 (−0.55–0.24)	0.440	0.95 (0.64–1.42)	0.820	0.36 (0.11–1.19)	0.093
**H6**	**0.52 (0.10–0.95)**	0.015 **	**1.87 (1.17–2.98)**	0.009 **	1.44 (0.68–3.03)	0.340

At least nominally significant association between haplotypes and TG/HDL-C ratio is highlighted in bold. ^a^ TG/HDL-C ratio cut-off for increased cardiometabolic risk (CMR) by Qurat et al. [21]. ^b^ TG/HDL-C ratio cut-off for extensive coronary disease (CD) risk by da Luz et al. [20]. ** Significant *p*-values with Bonferroni correction.

## References

[1] Torkamani A., Wineinger N.E., Topol E.J. (2018). The personal and clinical utility of polygenic risk scores. Nat. Rev. Genet..

[2] European Commission (2011). Communication from the Commission to the European Parliament, the Council, the European Economic and Social Committee, and the Committee of the Regions: An EU Framework for National Roma Integration Strategies up to 2020.

[3] Hungarian Central Statistical Office (2011). Population Census of Hungary in 2011.

[4] Council of Europe (2014). Estimates of Roma population in European Countries.

[5] Kosa K., Darago L., Adany R. (2011). Environmental survey of segregated habitats of Roma in Hungary: A way to be empowering and reliable in minority research. Eur. J. Public Health.

[6] Foldes M.E., Covaci A. (2012). Research on Roma health and access to healthcare: State of the art and future challenges. Int. J. Public Health.

[7] Kosa K., Adany R. (2007). Studying vulnerable populations: Lessons from the Roma minority. Epidemiology.

[8] Voko Z., Csepe P., Nemeth R., Kosa K., Kosa Z., Szeles G., Adany R. (2009). Does socioeconomic status fully mediate the effect of ethnicity on the health of Roma people in Hungary?. J. Epidemiol. Community Health.

[9] Zeljko H., Skaric-Juric T., Narancic N.S., Salihovic M.P., Klaric I.M., Barbalic M., Starcevic B., Lauc L.B., Janicijevic B. (2008). Traditional CVD risk factors and socio-economic deprivation in Roma minority population of Croatia. Coll Antropol..

[10] Dobranici M., Buzea A., Popescu R. (2012). The cardiovascular risk factors of the Roma (gypsies) people in Central-Eastern Europe: A review of the published literature. J. Med. Life.

[11] Zeljko H.M., Skaric-Juric T., Narancic N.S., Baresic A., Tomas Z., Petranovic M.Z., Milicic J., Salihovic M.P., Janicijevic B. (2013). Age trends in prevalence of cardiovascular risk factors in Roma minority population of Croatia. Econ. Hum. Biol..

[12] Weiss E., Japie C., Balahura A.M., Bartos D., Badila E. (2018). Cardiovascular risk factors in a Roma sample population from Romania. Rom. J. Intern. Med..

[13] Hernandez-PerezdelaBlanca M., Rebora-Mariano T., Ramirez-Robles R., Berna-Guisado C., Vides-Batanero M.C., Castro-Gomez J.A. (2014). Predictors of cardiovascular risk in a population of diabetic adults of Gypsy origin, in Granada. Bratislavske Lekarske Listy.

[14] Fedacko J., Pella D., Jarcuska P., Siegfried L., Janicko M., Veseliny E., Pella J., Sabol F., Jarcuska P., Marekova M. (2014). Prevalence of cardiovascular risk factors in relation to metabolic syndrome in the Roma population compared with the non-Roma population in the eastern part of Slovakia. Cent. Eur. J. Public Health.

[15] Kosa Z., Moravcsik-Kornyicki A., Dioszegi J., Roberts B., Szabo Z., Sandor J., Adany R. (2015). Prevalence of metabolic syndrome among Roma: A comparative health examination survey in Hungary. Eur. J. Public Health.

[16] NIH Consensus Conference (1993). Triglyceride, high-density lipoprotein, and coronary heart disease. NIH Consensus Development Panel on Triglyceride, High-Density Lipoprotein, and Coronary Heart Disease. JAMA.

[17] Tosheska Trajkovska K., Topuzovska S. (2017). High-density lipoprotein metabolism and reverse cholesterol transport: Strategies for raising HDL cholesterol. Anatol. J. Cardiol..

[18] Rader D.J., Hovingh G.K. (2014). HDL and cardiovascular disease. Lancet.

[19] Hokanson J.E., Austin M.A. (1996). Plasma triglyceride level is a risk factor for cardiovascular disease independent of high-density lipoprotein cholesterol level: A meta-analysis of population-based prospective studies. J. Cardiovasc. Risk.

[20] Da Luz P.L., Favarato D., Faria-Neto J.R., Lemos P., Chagas A.C. (2008). High ratio of triglycerides to HDL-cholesterol predicts extensive coronary disease. Clinics (Sao Paulo).

[21] Ul Ain Q., Asif N., Gilani M., Waqas Sheikh N., Akram A. (2017). To Determine Cutoff Value of Triglycerides to HDL Ratio in Cardio Vascular Risk Factors. Biochem. Anal. Biochem..

[22] Piko P., Fiatal S., Kosa Z., Sandor J., Adany R. (2017). Genetic factors exist behind the high prevalence of reduced high-density lipoprotein cholesterol levels in the Roma population. Atherosclerosis.

[23] Piko P., Fiatal S., Kosa Z., Sandor J., Adany R. (2019). Generalizability and applicability of results obtained from populations of European descent regarding the effect direction and size of HDL-C level-associated genetic variants to the Hungarian general and Roma populations. Gene.

[24] Zarkesh M., Daneshpour M.S., Faam B., Fallah M.S., Hosseinzadeh N., Guity K., Hosseinpanah F., Momenan A.A., Azizi F. (2012). Heritability of the metabolic syndrome and its components in the Tehran Lipid and Glucose Study (TLGS). Genet. Res. (Camb.).

[25] Todur S.P., Ashavaid T.F. (2013). Association of CETP and LIPC Gene Polymorphisms with HDL and LDL Sub-fraction Levels in a Group of Indian Subjects: A Cross-Sectional Study. Indian J. Clin. Biochem. IJCB.

[26] Wang J., Wang L.J., Zhong Y., Gu P., Shao J.Q., Jiang S.S., Gong J.B. (2013). CETP gene polymorphisms and risk of coronary atherosclerosis in a Chinese population. Lipids Health Dis..

[27] Clifford A.J., Rincon G., Owens J.E., Medrano J.F., Moshfegh A.J., Baer D.J., Novotny J.A. (2013). Single nucleotide polymorphisms in CETP, SLC46A1, SLC19A1, CD36, BCMO1, APOA5, and ABCA1 are significant predictors of plasma HDL in healthy adults. Lipids Health Dis..

[28] Thompson J.F., Lira M.E., Durham L.K., Clark R.W., Bamberger M.J., Milos P.M. (2003). Polymorphisms in the CETP gene and association with CETP mass and HDL levels. Atherosclerosis.

[29] Barter P. (2000). CETP and atherosclerosis. Arter. Thromb. Vasc. Biol..

[30] Carr M.C., Brunzell J.D., Deeb S.S. (2004). Ethnic differences in hepatic lipase and HDL in Japanese, black, and white Americans: Role of central obesity and LIPC polymorphisms. J. Lipid Res..

[31] Hodoglugil U., Williamson D.W., Mahley R.W. (2010). Polymorphisms in the hepatic lipase gene affect plasma HDL-cholesterol levels in a Turkish population. J. Lipid Res..

[32] Kathiresan S., Melander O., Guiducci C., Surti A., Burtt N.P., Rieder M.J., Cooper G.M., Roos C., Voight B.F., Havulinna A.S. (2008). Six new loci associated with blood low-density lipoprotein cholesterol, high-density lipoprotein cholesterol or triglycerides in humans. Nat. Genet..

[33] Daniels T.F., Killinger K.M., Michal J.J., Wright R.W., Jiang Z. (2009). Lipoproteins, cholesterol homeostasis and cardiac health. Int. J. Biol. Sci..

[34] Szeles G., Voko Z., Jenei T., Kardos L., Pocsai Z., Bajtay A., Papp E., Pasti G., Kosa Z., Molnar I. (2005). A preliminary evaluation of a health monitoring programme in Hungary. Eur. J. Public Health.

[35] Szigethy E., Szeles G., Horvath A., Hidvegi T., Jermendy G., Paragh G., Blasko G., Adany R., Voko Z. (2012). Epidemiology of the metabolic syndrome in Hungary. Public Health.

[36] Sole X., Guino E., Valls J., Iniesta R., Moreno V. (2006). SNPStats: A web tool for the analysis of association studies. Bioinformatics.

[37] Pers T.H., Timshel P., Hirschhorn J.N. (2015). SNPsnap: A Web-based tool for identification and annotation of matched SNPs. Bioinformatics.

[38] Wan K., Zhao J., Huang H., Zhang Q., Chen X., Zeng Z., Zhang L., Chen Y. (2015). The association between triglyceride/high-density lipoprotein cholesterol ratio and all-cause mortality in acute coronary syndrome after coronary revascularization. PLoS ONE.

[39] Radovica I., Fridmanis D., Vaivade I., Nikitina-Zake L., Klovins J. (2013). The association of common SNPs and haplotypes in CETP gene with HDL cholesterol levels in Latvian population. PLoS ONE.

[40] McCaskie P.A., Beilby J.P., Chapman C.M., Hung J., McQuillan B.M., Thompson P.L., Palmer L.J. (2007). Cholesteryl ester transfer protein gene haplotypes, plasma high-density lipoprotein levels and the risk of coronary heart disease. Hum. Genet..

[41] Feitosa M.F., Myers R.H., Pankow J.S., Province M.A., Borecki I.B. (2009). LIPC variants in the promoter and intron 1 modify HDL-C levels in a sex-specific fashion. Atherosclerosis.

[42] Guerra R., Wang J., Grundy S.M., Cohen J.C. (1997). A hepatic lipase (LIPC) allele associated with high plasma concentrations of high density lipoprotein cholesterol. Proc. Natl. Acad. Sci. USA.

[43] Lahoz C., Pena R., Mostaza J.M., Laguna F., Garcia-Iglesias M.F., Taboada M., Pinto X., Respuesta Ambulatoria a Pravastatina Study Group (2005). The -514C/T polymorphism of the hepatic lipase gene significantly modulates the HDL-cholesterol response to statin treatment. Atherosclerosis.

[44] Tall A.R. (1993). Plasma cholesteryl ester transfer protein. J. Lipid Res..

[45] Shrestha S., Wu B.J., Guiney L., Barter P.J., Rye K.A. (2018). Cholesteryl ester transfer protein and its inhibitors. J. Lipid Res..

[46] Winkelmann B.R., Hoffmann M.M., Nauck M., Kumar A.M., Nandabalan K., Judson R.S., Boehm B.O., Tall A.R., Ruano G., Marz W. (2003). Haplotypes of the cholesteryl ester transfer protein gene predict lipid-modifying response to statin therapy. Pharm. J..

[47] Bercovich D., Friedlander Y., Korem S., Houminer A., Hoffman A., Kleinberg L., Shochat C., Leitersdorf E., Meiner V. (2006). The association of common SNPs and haplotypes in the CETP and MDR1 genes with lipids response to fluvastatin in familial hypercholesterolemia. Atherosclerosis.

[48] Nair S.R. (2010). Personalized medicine: Striding from genes to medicines. Perspect. Clin. Res..

[49] Kang H.T., Yoon J.H., Kim J.Y., Ahn S.K., Linton J.A., Koh S.B., Kim J.K. (2012). The association between the ratio of triglyceride to HDL-C and insulin resistance according to waist circumference in a rural Korean population. Nutr. Metab. Cardiovasc. Dis..

[50] Zhou M., Zhu L., Cui X., Feng L., Zhao X., He S., Ping F., Li W., Li Y. (2016). The triglyceride to high-density lipoprotein cholesterol (TG/HDL-C) ratio as a predictor of insulin resistance but not of beta cell function in a Chinese population with different glucose tolerance status. Lipids Health Dis..

[51] Iwani N.A., Jalaludin M.Y., Zin R.M., Fuziah M.Z., Hong J.Y., Abqariyah Y., Mokhtar A.H., Wan Nazaimoon W.M. (2017). Triglyceride to HDL-C Ratio is Associated with Insulin Resistance in Overweight and Obese Children. Sci. Rep..

[52] Young K.A., Maturu A., Lorenzo C., Langefeld C.D., Wagenknecht L.E., Chen Y.I., Taylor K.D., Rotter J.I., Norris J.M., Rasouli N. (2019). The triglyceride to high-density lipoprotein cholesterol (TG/HDL-C) ratio as a predictor of insulin resistance, beta-cell function, and diabetes in Hispanics and African Americans. J. Diabetes Complicat..

[53] De Courten B.V., de Courten M., Hanson R.L., Zahorakova A., Egyenes H.P., Tataranni P.A., Bennett P.H., Vozar J. (2003). Higher prevalence of type 2 diabetes, metabolic syndrome and cardiovascular diseases in gypsies than in non-gypsies in Slovakia. Diabetes Res. Clin. Pract..

